# Sub-basin sea level budget analysis in the North Indian Ocean (2003–2024)

**DOI:** 10.1038/s41598-026-60856-5

**Published:** 2026-07-05

**Authors:** Ullas M. Pillai, Franck Eitel Kemgang Ghomsi, Ajith Joseph Kochuparampil, Roshin P. Raj, Ola M. Johannessen

**Affiliations:** 1 Nansen Environmental Research Centre (India), Kochi, 682506 India; 2 Faculty of Ocean Science and Technology, Kerala University of Fisheries and Ocean Studies, Kochi, 682506 India; 3 Centre for Earth Observation Science, University of Manitoba, Winnipeg, MB Canada; 4 Nansen-Tutu Centre for Marine Environmental Research, Department of Oceanography, University of Cape Town, Cape Town, South Africa; 5 National Institute of Cartography, P.O. Box 157, Yaoundé, Cameroon; 6 Nansen Environmental and Remote Sensing Center and Bjerknes Center for Climate Research, Bergen, Norway; 7 Nansen Scientific Society, Bergen, Norway

**Keywords:** North Indian Ocean, Sea level rise, Steric Sea level, GRACE gravimetry, Indian Ocean Dipole, Climate sciences, Ocean sciences

## Abstract

This study investigates sea level trends in the North Indian Ocean (NIO) from 2003 to 2024, quantifying the contributions of thermosteric, halosteric, and ocean mass components using satellite altimetry, observation-based gridded products, and GRACE gravimetry datasets. The NIO Sea level is rising at 4.83 ± 0.22 mm/yr, driven primarily by thermosteric sea level (2.21 ± 0.16 mm/yr) followed by ocean mass component (1.69 ± 0.09 mm/yr). Sub-basinal analysis reveals strong spatial heterogeneity, with the highest rates of sea level rise in the Western Bay of Bengal (5.19 ± 0.41 mm/yr) and the lowest in the Western Arabian Sea (4.29 ± 0.27 mm/yr). A distinct halosteric contrast exists, where freshening accelerates rise in the Bay of Bengal (up to 0.65 ± 0.08 mm/yr in the Eastern Bay), and increasing salinity suppresses it in the Arabian Sea (down to -1.12 ± 0.12 mm/yr in the western Arabian Sea). Furthermore, while western sub-basins are predominantly steric-driven, eastern sub-basins of Bay of Bengal and Equatorial Indian Ocean exhibit anomalously high GRACE-derived mass contributions (> 3 mm/yr), likely influenced by post-seismic crustal adjustments from the 2004 Sumatra-Andaman earthquake. Interannual variability closely tracks steric changes modulated by ENSO and the Indian Ocean Dipole (IOD), triggering basin-specific responses via wind anomalies and long-period waves. Further analysis of tropical SST indices reveals that the Western Tropical Indian Ocean SST index closely mirrors the spatial correlation structures of the Dipole Mode Index, and may serve as a useful indicator of regional sea level variability, particularly over the Equatorial Indian Ocean and Bay of Bengal. Ultimately, unlike the predominantly mass-driven global trends where both mass and steric contributions are of comparable magnitude, the NIO remains uniquely steric-dominated and the sea level budget leaves a residual of 0.98 mm/yr (~ 20% of the total trend), which, while comparable to uncertainties in individual components, highlights remaining challenges in fully closing the regional sea level budget.

**Supplementary Information:**

The online version contains supplementary material available at https://doi.org/10.1038/s41598-026-60856-5.

## Introduction

In recent decades, sea level research has gained critical importance. Globally, nearly 40% of the population resides within 100 km of coastlines, with about 10% inhabiting vulnerable low-lying areas, including Small Island States that face increasing risks from coastal flooding, erosion, saltwater intrusion, and ecosystem degradation resulting from the rising sea level^1^. Current sea level rise (SLR), along with its observed acceleration as measured by satellite altimetry^2,3^, is largely driven by human-caused global warming. The thermal expansion from ocean warming and mass addition from land ice melt are considered to be the primary components of sea level rise which are directly linked to anthropogenic climate change. The Global Mean Sea Level (GMSL) has been designated by the World Meteorological Organization (WMO) as one of the seven essential climate change indicators, following recommendations from the Global Climate Observing System^4^. The consequences of rising seas extend beyond threats to coastal urban centres, potentially transforming entire shorelines, and leading to permanent inundation^5^. These physical changes have fundamentally altered coastal ecosystems and tidal dynamics, creating cascading environmental and societal impacts^6–8^.

Changes in GMSL arise from two primary physical processes. The first is steric change, resulting from variations in ocean density due to temperature and salinity changes. The second is barystatic (or ocean mass) change, driven by the addition of water by ice loss from glaciers and ice sheets of Greenland and Antarctica, as well as changes in terrestrial water storage, such as groundwater depletion or reservoir retention. According to Thompson et al.^9^, the updated global mean sea level trend from satellite altimetry for 1993–2022 is 3.4 ± 0.4 mm/yr, while the global mean ocean mass trend for 2005–2022 is 2.1 ± 0.4 mm/yr. While ocean mass addition remains the dominant contributor to the recent global sea-level rise, followed by steric contributions^7,10–12^, these processes are fundamentally driven by climatic factors, including modifications to atmospheric and oceanic conditions^13^. An approximate equilibrium between Indian Ocean warming and Atlantic Ocean cooling was observed during 2004–2010 which accounted for the decelerated rate of observed global steric sea level rise during those years^14^. Recent observations show that ice sheet melt is accelerating, particularly in Greenland^15^. While these factors drive the long-term trend, research has established that interannual GMSL fluctuations are predominantly influenced by natural climate variability. The El Niño-Southern Oscillation (ENSO) and the Pacific Decadal Oscillation (PDO) are the primary drivers of these short-term anomalies^15–17^.

At a regional scale, satellite altimetry data demonstrate that SLR often varies significantly from the global mean (e.g., Raj et al.^19^). Regional sea-level variations at decadal scales reflect global mean rise plus spatial anomalies from steric effects, ocean dynamics, atmospheric forcing, and solid Earth responses to mass redistribution^7,20^. Precise monitoring of these regional variations and their drivers is critical for detection-attribution studies, especially when it comes to identifying when anthropogenic signals appear in different areas^21,22^. Locally, the most relevant metric for assessing societal impacts is the change in sea level relative to the land surface. Coastal sea-level trends often differ from open-ocean trends due to the influence of small-scale processes, such as winds, currents, and vertical land motion (isostatic adjustments), that modify the broader regional and global signals^23^. Palanisamy et al.^24^ distinguished “climate-related” sea level change (global mean plus regional pattern) from “total relative” sea level (including vertical land motion), showing that subsidence can locally amplify Indian Ocean coastal sea level change well beyond the climatic signal. The consequences of SLR are evident worldwide in coastal regions, with increasingly persistent effects^7,25^.

The launch of satellite altimetry missions in 1992, including TOPEX/Poseidon and its successors, revolutionized sea-level monitoring by enabling precise measurements of absolute sea-level change. To isolate the components driving these observed changes, space gravimetry missions were deployed. The Gravity Recovery and Climate Experiment (GRACE, 2002–2017) and GRACE Follow-On mission (2018-present), complement altimetry by quantifying ocean mass redistribution through gravity anomaly measurements^26^. Together, these satellite systems have dramatically improved the separation and estimation of individual sea-level components (steric and barystatic) with unprecedented accuracy. Earlier studies in the North Indian Ocean (NIO) has already exploited such multi-sensor combinations, for example, Ghosh et al.^27^ combined altimetry, GRACE, and Argo in the Bay of Bengal and showed that the sum of steric and mass components was broadly consistent with altimetric sea level within uncertainties, while highlighting regional discrepancies linked to sparse Argo coverage and GRACE noise. More recently, Ghomsi et al.^7^ applied a similar framework to Africa’s Large Marine Ecosystems (LMEs) and found that mass contributions now account for over 80% of total sea level rise in those regions, a stark contrast with the steric-dominated signal reported for the tropical Indian Ocean.

NIO exhibits complex oceanographic dynamics due to its unique geographical constraints and seasonal forcing due to the monsoon system. As a semi-enclosed ocean bounded by land on the northern side, it experiences complete seasonal current reversals driven by monsoon wind patterns^26,28–31^. The ocean features two distinct sub-seas, the Arabian Sea in the west, which is characterized by high-salinity water outflows from the Persian Gulf and the Red Sea, and the Bay of Bengal (BoB) in the east, which is dominated by strong freshwater input from major river systems. The sea level in the North Indian Ocean is dominated by thermosteric changes, which have accelerated in recent decades^32,33^. Swapna et al.^32^ identified a mechanistic link between multidecadal weakening of the Indian summer monsoon circulation and thermosteric sea level rise in the NIO, demonstrating that reduced southward heat transport via the Cross-Equatorial Cell (CEC) leads to enhanced heat retention and thermosteric expansion, particularly in the Arabian Sea. Furthermore, Srinivasu et al.^34^ documented a distinct decadal reversal in NIO sea level trends around 2003, with sea level falling during 1993–2003 and rising sharply during 2004–2013, attributing this reversal to changes in surface turbulent heat flux and meridional heat transport driven by decadal wind variability. Salim et al.^35^ showed that, over the broader tropical Indian Ocean during 1993–2007, steric processes accounted for about 35% of long-term sea level change, with a particularly strong steric control (~ 72%) in the south tropical Indian Ocean but weaker and even negative steric contributions in parts of the north, underlining strong meridional contrasts. Interannual sea-level variability in the Indian Ocean is primarily driven by major climate modes, particularly the El Niño-Southern Oscillation (ENSO) and Indian Ocean Dipole (IOD)^36^. Nidheesh et al.^37^ demonstrated that decadal sea level variability in the Indo-Pacific is driven by wind stress variations that are relatively independent between the Pacific and Indian Ocean basins, with thermosteric changes almost entirely dominating decadal fluctuations, while cautioning that long-term trend estimates from forced ocean models are sensitive to wind product uncertainties. However, in coastal regions, halosteric variations induced by fluctuating river discharge and enhanced precipitation can actively influence decadal-scale sea level variability^38,39^. Palanisamy et al.^24^ further demonstrated, using a 60-year reconstruction (1950–2009), that steric changes and IOD-related variability dominate the climatic component of Indian Ocean Sea level and that regional patterns such as the east-west trend increase below ~ 15°S and evolve on multidecadal time scales. Rising sea levels due to climate change pose significant threats to a coastal nation like India, where approximately 420 million of its 1.4 billion inhabitants live in coastal regions^40^. India’s eastern coastline faces increased vulnerability to sea-level rise, and the risk is compounded by intensifying cyclonic activity^1^. Parekh et al.^36^ reported that the BoB exhibits substantially higher sea level rise rates (up to 8.8 mm/yr at Charchanga, Bangladesh) compared to the Arabian Sea, with steric contributions dominating mass-driven changes by a factor of two, highlighting the regional heterogeneity that our study aims to characterize at sub-basin scales.

**Fig. 1:**
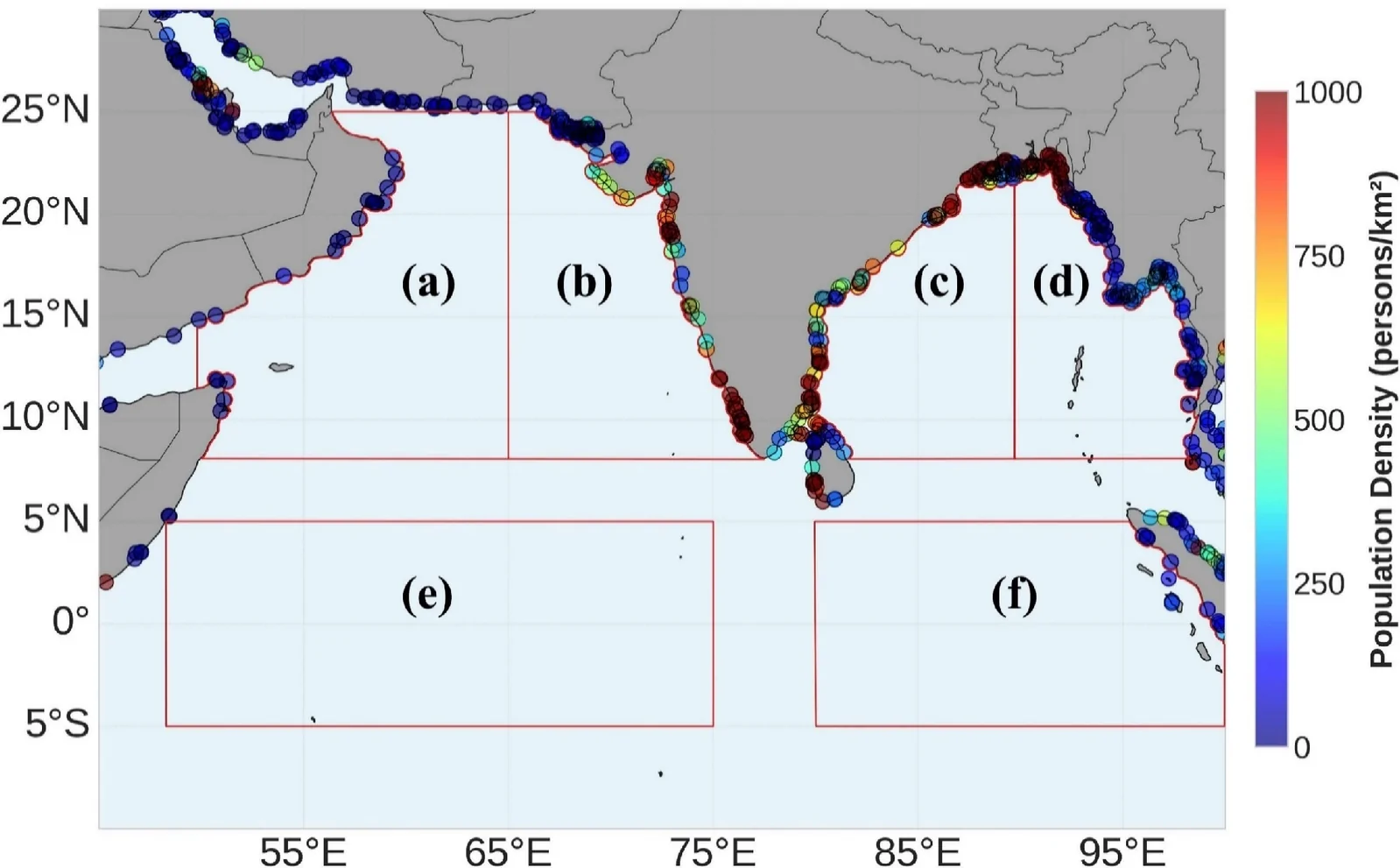
Study area showing the North Indian Ocean (NIO; 45°E-100°E; 10°S-30°N) and its sub-basins considered in this study: **(a)** Western Arabian Sea (WAS; 50°E-65°E, 8°N-25°N), **(b)** Eastern Arabian Sea (EAS; 65°E-78°E, 8°N-25°N), **(c)** Western Bay of Bengal (WBoB; 80°E-90°E, 8°N-22°N), **(d)** Eastern Bay of Bengal (EBoB; 90°E-100°E, 8°N-22°N), **(e)** Western Equatorial Indian Ocean (WEIO; 48°E-75°E, 5°S-5°N), and **(f)** Eastern Equatorial Indian Ocean (EEIO; 78°E-100°E, 5°S-5°N). The coloured circles along the coastal boundaries shows the population density within 20 kms from the coast that are less than 10 m above sea level.

This study assesses the individual contributions of steric (thermosteric and halosteric effects) and ocean mass components to sea-level variations across distinct sub-basins of the North Indian Ocean (see Fig. 1) during the GRACE mission era 2003–2024 and provides an updated estimates on recent regional sea level trends and variability by providing a spatial and temporal analysis of these contributions. By extending earlier basin-scale work over the tropical Indian Ocean^35^ and longer-term reconstructions^24^ into the GRACE/altimetry era with higher-resolution ARMOR3D fields, our study focuses specifically on sub-basin differences within the NIO and quantifies how the relative importance of thermosteric, halosteric, and mass terms has evolved in the most recent decades. Our study period (2003–2024) corresponds to the period of accelerated NIO sea level rise, identified by Srinivasu et al.^34^ and extends the multidecadal analysis of Swapna et al.^32^, allowing us to assess whether the thermosteric dominance and monsoon-driven mechanisms they identified continue to characterize the most recent decades. We also address sea level contributions from glacial isostatic adjustment (GIA), which is a long-term process whereby the Earth’s crust rises or subsides as it responds to the residual effects of ice sheet loading from the past^41^ including the contribution from atmospheric forcing. Furthermore, this study evaluates how climate modes, such as ENSO and Indian Ocean Dipole (IOD), influence interannual sea-level variability in each sub-basin during the altimetry period from 1993 to 2024. This allows us to distinguish natural variabilities from long-term anthropogenic signals. The paper is structured as follows: Sect. "Data and methods" describes the data and methods; Sect. "Results and discussion" presents the results and discussion; and Sect. "Summary and conclusions" provides the summary and conclusions.

## Data and methods

### Altimetry sea level data

Daily gridded altimetric sea level anomaly (SLA) data with a spatial resolution of 1/8° x 1/8° over the NIO from 1993 to 2024 (https://doi.org/10.48670/moi-00148, accessed June 2026) were obtained from the Copernicus Marine Environment Monitoring Service (CMEMS). This dataset was processed using the DUACS multi-mission altimeter processing system, which involves merging observations from several altimeter missions to ensure data reliability. Additionally, all standard corrections, including sea state bias, instrumental drift, and dynamic atmospheric correction, were applied to the dataset^42^. For each grid point, the monthly mean of SLA was calculated from the daily values. Our approach is consistent with previous Indian Ocean studies using multi-mission merged altimetry^27,35^ and with recent applications to African LMEs^7^, ensuring robustness of regional sea level trends.

### Estimation of steric sea level components

An ensemble approach is adopted to estimate the steric sea level components using monthly mean temperature and salinity profiles from the ARMOR3D dataset, distributed by the CMEMS (https://doi.org/10.48670/moi-00052, accessed June 2026), and the Institute of Atmospheric Physics (IAP) dataset (http://www.ocean.iap.ac.cn/, accessed June 2026). The ARMOR3D dataset is a combination of satellite and in-situ observations that provide steric sea level estimates^43,44^ and it is available with a spatial resolution of 0.125° × 0.125° and 50 vertical levels. The IAP dataset consists of Version 4.2 monthly gridded ocean temperature and Version 2 monthly gridded salinity fields at 1° × 1° horizontal resolution and 41 vertical levels. These products are derived from bias-corrected in situ observations from the World Ocean Database, supplemented by IAP observations, with model simulations guiding the gridding process^45^. The two datasets are regridded to 0.25° × 0.25° horizontal resolution.

Steric sea level anomaly (SSLA) was computed by vertically integrating density anomalies and then decomposing them into thermosteric (TSLA) and halosteric (HSLA) components using established methods^10,46,47^ as shown in Eq. (1)

1 $$\:SSLA=TSLA+HSLA=\:\frac{-1}{{\rho\:}_{0}}{\int\:}_{-H}^{0}\varDelta\:\rho\:dz\:=-{\int\:}_{-H}^{0}\left(\alpha\:\varDelta\:T-\beta\:\varDelta\:S\right)dz$$

Here, $\:{\rho\:}_{0}$ is the reference density (1025 kg/m³), and * z* represents depth. $\:\varDelta\:\rho\:$, $\:\varDelta\:T$, and $\:\varDelta\:S$ denote anomalies in density, temperature, and salinity, respectively, relative to their climatological means at each depth level. The coefficients $\:\alpha\:$ (thermal expansion) and $\:\beta\:$ (haline contraction) were derived from monthly temperature and salinity fields using the Thermodynamic Equation of Sea Water − 2010 (TEOS-10)^48^ and the Gibbs Sea Water (GSW) Oceanographic Toolbox^49^. The reference depth * H* is defined as 2000 m, reflecting the depth limit of Argo profiling floats, beyond which observational coverage is sparse^50^.

### Dynamic atmospheric correction (DAC), glacial isostatic adjustment (GIA), and ocean mass component

The contribution of changing atmospheric forcing to the sea level variability can be assessed using the Dynamic Atmospheric Correction dataset. This dataset, provided by Aviso (https://www.aviso.altimetry.fr/en/data/products/auxiliary-products, accessed June 2026), is updated every six hours and has a spatial resolution of 0.25°×0.25°, starting from 1992. Contributions from the ocean mass component were estimated using the ensemble mean of the RL06 GRACE/GRACE-FO mascon solutions from three GRACE Science Data Service Centers, namely the Center for Space Research^51^(CSR, http://www2.csr.utexas.edu/grace), the Jet Propulsion Laboratory^52^(JPL, https://podaac.jpl.nasa.gov), and the NASA Goddard Space Flight Center^53^(GSFC, https://earth.gsfc.nasa.gov/geo/data/grace-mascons). To ensure consistency among the datasets and comparability with satellite altimetry-derived sea level anomalies, the mean GAD signal, which contains the high-frequency non-tidal atmospheric and oceanic mass variations, was removed from the CSR and JPL datasets, as it is already excluded from the GSFC product. The JPL dataset additionally incorporates a Coastal Resolution Improvement (CRI) filter to reduce signal leakage across coastlines. The mascon solutions are available from April 2002 onward and are regridded to a 0.25° × 0.25° resolution from their sampled grid resolution for this study. Corrections for glacial isostatic adjustment (GIA) are applied using the ICE6G-D model^54^. Following the approach of Ghosh et al.^27^ over the BoB, Palanisamy et al.^24^ over the wider Indian Ocean, and Ghomsi et al.^7^ over African coastal waters, GIA corrections are essential to ensure consistency between GRACE-based mass trends, steric components and altimetric sea level, especially in regions where in situ measurements of vertical land motion are sparse.

To understand the role of climatic influence on sea level, we considered the variability of sea level during ENSO and IOD years and used the Relative Oceanic Niño Index (RONI) and the Dipole Mode Index (DMI), both obtained from the Physical Sciences Laboratory of NOAA (https://psl.noaa.gov/data/timeseries/month, accessed June, 2026). The RONI adjusts the standard ONI for the warming trend in tropical Pacific SSTs, making it more appropriate for long-term sea level variability studies. Here RONI is used throughout and referred to as RONI in all figures. Additionally, the Western Tropical Indian Ocean (WTIO) and Southeastern Tropical Indian Ocean (SETIO) sea surface temperature (SST) indices were obtained from https://stateoftheocean.osmc.noaa.gov/sur/ind/setio.php. The DMI is the difference between the WTIO and SETIO SST indices.

We applied a non-parametric Mann-Kendall test, which is robust in detecting monotonic trends in the presence of noise and non-normality, in order to statistically assess the spatial trends for significance at a 95% confidence level for the various sea level contributions, following the methodologies used in previous studies^6,10^. To quantify the magnitude of these trends, we used Sen’s slope estimator, which is another non-parametric method used along with Mann-Kendall test, that remains unaffected by data outliers^55^. Unlike the parametric Ordinary Least Square (OLS) method, Mann-Kendall test effectively detects both linear and non-linear trends and exhibits low sensitivity to abrupt breaks within a time series^56^. Subsequently, the spatial correlations between the interannual sea level variability and different climate modes were evaluated. The seasonal signal was removed by calculating the long-term monthly climatology across the study period (2003–2024) and subtracting it from each corresponding individual month to yield the deseasonalized anomalies. For the comparison of trends, all the datasets are arranged according to the time period of the GRACE dataset, which has missing time steps of totaling approximately 14% of the data, as a result of the inter-mission gap and battery issues. However, no interpolation was applied across the missing periods; instead, all datasets were masked to the same available GRACE epochs, and the Mann-Kendall test was applied to the resulting irregular time series ensuring that any differences between the estimates are not due to gaps in the GRACE dataset.

## Results and discussion

### Spatial trends of various contributors to sea level change during the GRACE/GRACE-FO mission era

**Fig. 2:**
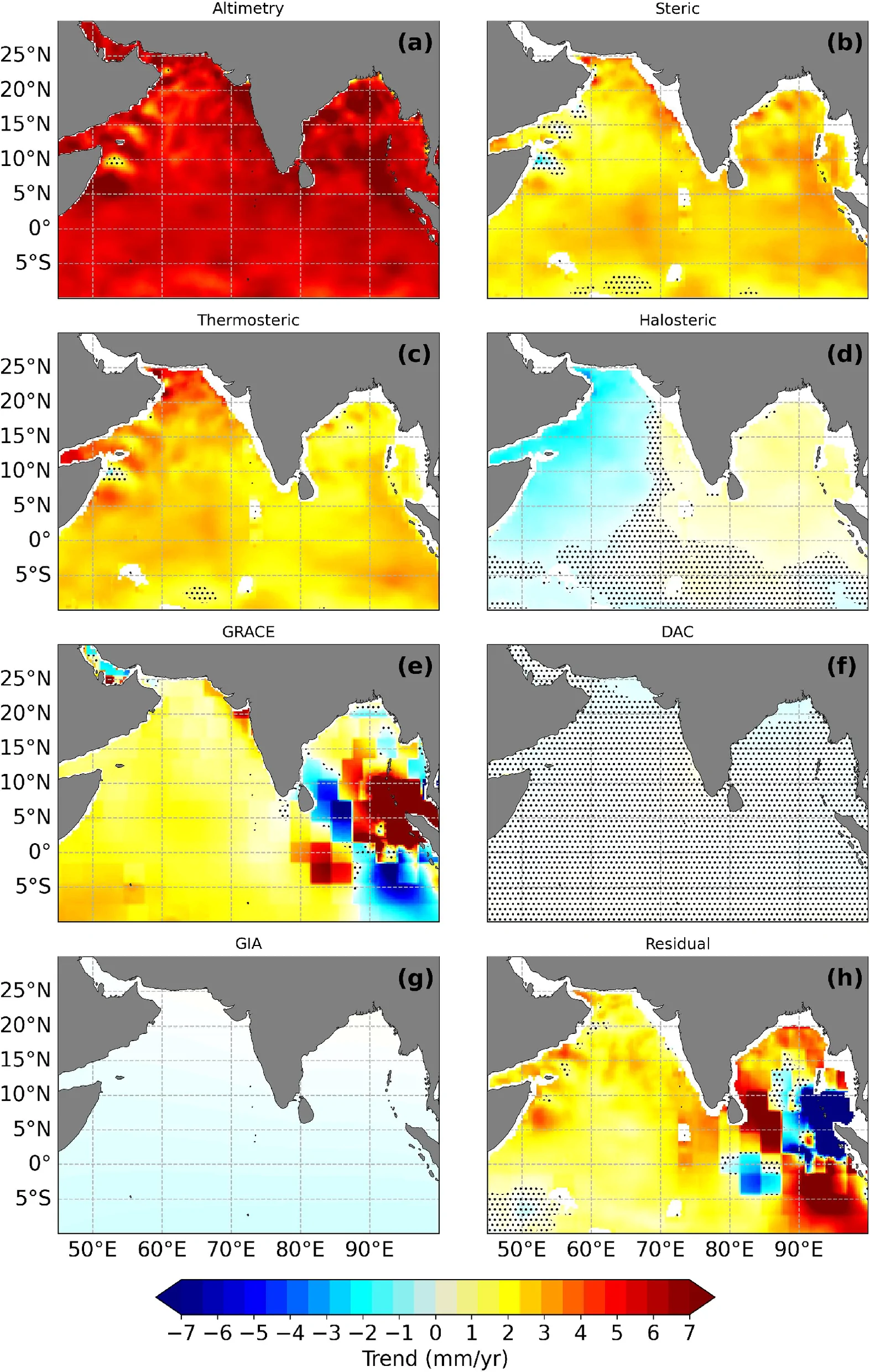
Spatial Mann-Kendall trends of SLA (mm/yr) from 2003 to 2024 for different components of sea level: **(a)** Satellite Altimetry, **(b)** Steric, **(c)** Thermosteric, **(d)** Halosteric, **(e)** GRACE Ocean mass, **(f)** DAC, **(g)** GIA, and **(h)** Residual. Regions where trends are not statistically significant at the 95% confidence level are stippled, except for GIA, for which only the integrated linear trend is available. The steric components constitute the ensemble mean from ARMOR3D and IAP datasets whereas GRACE ocean mass component is obtained from the ensemble mean of JPL, GSFC and CSR datasets.

This section discusses the major contributing factors and their respective roles in shaping the total sea level trend. Figure 2 illustrates the spatial trends of various components contributing to total sea level variability over the North Indian Ocean during the period 2003 to the end of 2024. Altimetric SLA (Fig. 2a) shows strong positive trends, exceeding 7 mm/yr in several regions, especially in the north and south of the BoB, along the western coast of India between 16°N to 21°N and near the somali coast between 5°N to 8°N. Trends near the Somali coast may partly reflect the influence of the Somali Current and associated mesoscale eddy systems such as the Great Whirl, which can alias into long-term trend estimates. The higher trends in BoB are broadly consistent with earlier altimetric estimates^27^ of 4.36 mm/yr during 2004–2010, but our comparatively longer time span and higher-resolution inputs reveal stronger accelerations, with an averaged trend of 5.11 ± 0.49 mm/yr. Coastal areas of India exhibit consistent rising sea level trends, ranging from approximately 1.65 mm/yr off the northern BoB coast near 21°N, 87°E to 7.72 mm/yr off the southwestern coast near 20°N, 70°E. Non-significant or weakly increasing trends (below 1.5 mm/yr) are observed primarily in the western Arabian Sea, near the Somali coast. The spatial average of the altimetric sea level trend over the Arabian Gulf exceeds 4.3 mm/yr, which closely aligns with the 4.29 mm/yr mean trend previously documented by Al-Subhi & Abdulla^57^ for the period from 2000 to 2021. In the equatorial Indian Ocean sub-basins, the eastern sub-basin shows higher averaged trends (~ 4.85 mm/yr), with peaks nearing 6.85 mm/yr along the Indonesian coastline.

The steric components, comprising total steric (Fig. 2b), thermosteric, depicting the contribution of thermal expansion to sea level (Fig. 2c), and halosteric, depicting the sea level variation due to salinity changes (Fig. 2d), are computed for the upper 2000 m of the water column. While secondary to upper-ocean drivers, the steric signal below 900 m is noteworthy and is anticipated to grow over time as the abyssal ocean continues to shift^58^. The total steric component shares a spatial similarity with the thermosteric pattern than the halosteric in most of the NIO, reinforcing that ocean temperature is the dominant driver of steric sea level variability. This thermosteric dominance is consistent with the findings of Nidheesh et al.^37^, who demonstrated that decadal sea level variations in the Indo-Pacific are almost entirely thermosteric, with halosteric contributions significant only in localized regions. However, in the western Arabian Sea (WAS), the pronounced negative halosteric trend (Fig. 2d) partially offsets the positive thermosteric trend (Fig. 2c), thereby reducing the net steric sea level trend. The thermosteric trends are more prominent in the Arabian Sea compared to the Bay of Bengal. The enhanced thermosteric signal in the Arabian Sea aligns with the mechanism proposed by Swapna et al.^32^, who showed that weakening monsoon circulation reduces coastal upwelling off Somalia and Arabia, leading to increased heat retention and thermosteric sea level rise in this region. Notably, the western Arabian Sea, particularly around the Gulf of Oman and Gulf of Aden, shows a dipole pattern in its steric components, with strong positive thermosteric trends and strong negative halosteric trends. These features are influenced by the warm, saline outflows from the Persian Gulf and the Red Sea, respectively, which alter local temperature and salinity profiles. Halosteric changes (Fig. 2d) exhibit significant trends in both the Arabian Sea (−0.71 ± 0.11 mm/yr) and the BoB (0.60 ± 0.07 mm/yr), although their magnitude is generally lower than thermosteric contributions. Nevertheless, in the northeastern BoB, eastern equatorial Indian Ocean, and off India’s western coast (east of 70°E, north of 16°N), halosteric trends reach up to ~ 1 mm/yr, indicating localized salinity-driven SLR. This pattern of strong thermosteric control with secondary halosteric modulation is consistent with the CEOF-based decomposition of SSH and steric height found by Salim et al.^35^, who reported close coherence between the leading steric and SSH modes over the tropical Indian Ocean. It also contrasts markedly with African LMEs, where Ghomsi et al.^7^ found that the halosteric contribution is typically negative or negligible while mass contributions dominate the sea level budget. Importantly, the halosteric patterns we observe, negative in the Arabian Sea and positive in the BoB, are consistent with the contrasting salinity regimes described by Jyoti et al.^59^ for the South Indian Ocean, where halosteric contributions account for approximately 40% of the accelerated sea level rise during 2000 to 2015, primarily driven by Indonesian Throughflow transport of fresher Pacific waters.

The GRACE satellite mission offers complementary insights by quantifying the mass-driven component of sea level (Fig. 2e). The Arabian Sea, in particular, exhibits positive trends in ocean mass component, with an area-averaged trend of 1.08 mm/yr. In contrast, the eastern NIO displays a complex mix of strong positive and negative trends. These spatial anomalies have been linked to crustal adjustments following the 2004 Sumatra-Andaman earthquake, which induced significant geophysical changes in the regional geoid and ocean mass distribution^60–62^. Importantly, the 2004 earthquake introduced a near-instantaneous step change in the regional geoid detected by GRACE. Fitting a linear trend over 2003–2024 will therefore systematically absorb part of this step change as an apparent positive trend in the mass component, inflating the estimated ocean mass contribution for the eastern sub-basins. This is a fundamental limitation of linear trend analysis in earthquake-affected regions and should be considered when interpreting the EEIO and EBoB mass budgets. Nonetheless, GRACE-derived estimates must be interpreted with caution. The mission’s coarse spatial resolution increases the risk of signal leakage, especially from land into adjacent coastal ocean regions, potentially distorting the actual magnitude and spatial structure of mass changes^63^. Numerous studies have reported challenges in estimating ocean mass using GRACE data, particularly for specific ocean basins like Indian Ocean^64^, where the signal leakage and measurement uncertainties are significant. Also, the non-steric sea level variability induced by the earthquake, while detectable through satellite gravimetry as geoid height variations, remains indistinguishable in satellite altimetry because of the overwhelming influence of steric sea level variations^65^. Ghosh et al.^27^ similarly reported poor regional agreement between GRACE-based mass trends and the difference between altimetry and Argo-based steric sea level in the BoB, underlining that GRACE-derived basin-scale mass budgets over the north Indian Ocean currently remain more uncertain than global estimates. Despite these limitations, GRACE data remain valuable for identifying large-scale ocean mass variability, particularly when integrated with steric and altimetric observations to assess the full spectrum of sea level change drivers.

The DAC is a correction applied to account for the high-frequency sea level variations caused by changing atmospheric pressure. The DAC (Fig. 2f) shows negligible trends across the NIO, with values nearly 0.01 mm/yr and lacking statistical significance. This confirms that atmospheric pressure-related effects, as accounted for SLR by the DAC, have minimal influence on long-term sea level trends in the region and do not show any spatial variability. The GIA dataset from Peltier et al.^54^ is presented as an integrated linear trend for the full period (Fig. 2g); as in Palanisamy et al.^24^ and Ghomsi et al.^7^. This prevents a formal statistical assessment of trend significance but provides a necessary correction for long-term solid-Earth adjustment.

Figure 2h shows the residual SLA, estimated by subtracting the steric, ocean mass component, and GIA from the altimetry SLA, capturing contributions of unmodeled signals which may originate from various sources, including deep-ocean contributions below 2000 m depth, drifts or biases in the models used for steric and barystatic components, and effects of small-scale ocean dynamic processes such as wind-driven currents. The residual sea level accounts for about 20.2% of the observed sea level trend across the NIO. However, the residual SLA likely reflects a combination of unresolved processes such as vertical land motion, and potentially uncorrected tidal effects, which are not isolated in this study. Based on published global estimates^66,67^, the contribution of the deep ocean (below 2000 m) to steric sea level is of order 0.1–0.2 mm/yr and therefore insufficient to explain the full residual. Vertical land motion, which is not corrected for in this study, may contribute significantly in the EAS and northern BoB where subsidence from sediment loading and groundwater extraction has been documented. Similar residual discrepancies between the sum of steric and mass-driven components with altimetric sea level have been reported previously in the BoB^27^ and at the scale of the tropical Indian Ocean^35^, and have been attributed to sparse in situ coverage of deep ocean, GRACE noise, and land-ocean signal leakage. In light of these uncertainties, the thermosteric component (Fig. 2c) emerges as the most consistent and physically robust driver of sea level trends in the NIO, confirming the conclusions of Swapna et al.^68^ and Jyoti et al.^69^.

The trend estimated using Mann-Kendall test as shown in Fig. 2, observed for the six sub-basins of the NIO, given in Table 1, highlight notable differences in sea level change dynamics across the Arabian Sea, BoB, and Equatorial Indian Ocean (EIO). Comparison of trends using the OLS method shown in Table S1 of the Supplementary material proves that the sea level trends are stable, monotonic, and not being driven or distorted by extreme single-year outliers since there is an exceptional agreement between the trends estimated using the OLS and the Mann-Kendall methods. From Table 1, each region exhibits distinct contributions from steric, mass-driven, and residual components. Based on satellite altimetry, the BoB records the highest basin-wide, area-averaged sea level trend of 5.11 ± 0.49 mm/yr, followed by the EIO (4.93 ± 0.30 mm/yr) and the Arabian Sea (4.55 ± 0.22 mm/yr). These basin-scale trends are computed directly from area-averaged sea level (not shown in the table). The total steric contribution in the Arabian Sea (1.73 ± 0.19 mm/yr) is relatively lower than in the other two basins (2.39 ± 0.34 mm/yr in BoB and 2.31 ± 0.28 mm/yr in the EIO). However, when the steric components are considered separately, the thermosteric trend in the Arabian Sea is the highest among all regions (2.40 ± 0.23 mm/yr), indicating a strong influence of ocean warming on the sea level rise. This pronounced thermosteric signal in the Arabian Sea supports the results of Swapna et al.^32^ that weakened summer monsoon circulation has reduced southward heat export via the Cross-Equatorial Cell, resulting in enhanced heat storage and thermosteric expansion in the NIO, particularly in regions of suppressed upwelling. In contrast, the halosteric component in the Arabian Sea shows a significant negative trend of −0.71 ± 0.11 mm/yr, which reduces the net steric trend to 1.73 ± 0.19 mm/yr. This salinity-driven contraction offsets much of the thermosteric expansion, thereby moderating the overall steric SLR in the region.

**Table 1:** Area-averaged trends (Mann-Kendall) in sea level and its components across the North Indian Ocean (NIO) and its sub-basins from 2003 to 2024 (units in mm/yr). The Total SLA consists of the entire altimetry trend from 2003 to 2024, whereas for the remaining components, the sea level trend corresponding to the time period of GRACE dataset (including the missing periods) from 2003 to 2024 is considered. (*Non-significant trends).

Region	Sub-basin	Total Altimetry SLA (mm/yr)	Altimetry SLA (mm/yr)	Steric (mm/yr)	Thermosteric (mm/yr)	Halosteric (mm/yr)	GRACE (mm/yr)	Residual (mm/yr)
Arabian Sea	WAS	4.12 ± 0.61	4.29 ± 0.27	1.47 ± 0.26	2.55 ± 0.25	−1.12 ± 0.12	1.10 ± 0.12	1.66 ± 0.15
	EAS	4.64 ± 0.69	4.80 ± 0.28	2.08 ± 0.21	2.15 ± 0.22	−0.15 ± 0.15*	1.06 ± 0.14	1.69 ± 0.17
Bay of Bengal	WBoB	4.96 ± 0.80	5.19 ± 0.41	2.34 ± 0.34	1.79 ± 0.31	0.57 ± 0.07	0.90 ± 0.14	2.07 ± 0.26
	EBOB	4.85 ± 0.99	4.93 ± 0.56	2.36 ± 0.46	1.70 ± 0.37	0.65 ± 0.08	3.04 ± 0.23	−1.47 ± 0.24
Equatorial Indian Ocean	WEIO	4.41 ± 0.85	4.45 ± 0.42	2.09 ± 0.36	2.07 ± 0.33	−0.17 ± 0.11	1.39 ± 0.10	1.23 ± 0.13
	EEIO	4.85 ± 0.93	4.95 ± 0.51	2.34 ± 0.41	2.10 ± 0.42	0.29 ± 0.07	3.42 ± 0.11	−0.52 ± 0.18
NIO	NIO	4.66 ± 0.64	4.83 ± 0.22	2.23 ± 0.18	2.21 ± 0.16	−0.10 ± 0.05	1.69 ± 0.09	0.98 ± 0.09

Across the Arabian Sea sub-basins and the western BoB, the residual sea level, which represents the remainder of the SLR derived from altimetric signals that is not accounted for by steric, ocean mass components and GIA effects, contributes substantially to the total trend. Our estimation of residual trends is subject to increased uncertainty in the Eastern Arabian Sea (EAS) and Eastern Bay of Bengal (EBoB). This limitation arises from the spatial constraint imposed by the depth-integration used to define the steric component, which effectively masks the contribution of steric variability over the continental slope to the shelf which is shallower and has limited observations from Argo floats. Part of these residuals may also reflect vertical land motion, as shown for certain Indian Ocean coasts and islands by Palanisamy et al.^24^ and for African deltaic regions by Ghomsi et al.^7^, although our study does not explicitly separate this contribution.

Even after accounting for the GRACE-derived ocean mass component, the Arabian Sea retain strong basin-scale residual trends of 1.66 mm/yr compared to the BoB (0.72 mm/yr) (Fig. 2h). A study by Unnikrishnan & Antony^70^ mentioned the increase in mean sea level at the head of the Bay, possibly due to subsidence of the Ganga-Brahmaputra delta, which further results in increased frequency of extreme sea levels. In comparison, regionally averaged residual trend of the EIO is nearly 0.57 mm/yr, mainly due to uncertainties related to the GRACE data over the Eastern Equatorial Indian Ocean. Although the thermosteric component is one of the dominant drivers of sea level in most of these basins, the ocean mass component measured from GRACE gravimetry also contributes significantly to the total sea level trend, as observed in the BoB and the EIO. Apart from these, the residual trends observed in the sub-basins also reveal the influence of some non-steric sea level components that need to be accounted for in future studies. This underscores the regional complexity of SLR in the Indian Ocean. When expressed as a fraction of the total NIO trend, our results imply that steric processes explain roughly 46% of recent sea level rise, which is larger than the basin-wide 35% reported by Salim et al.^35^ for the 1993–2007 period and substantially larger than the ~ 20% steric contribution found for African LMEs by Ghomsi et al.^7^. This contrast highlights that the NIO is unusually steric-dominated compared to other regional seas, where mass-driven contributions from ice melt and terrestrial water storage changes have become increasingly prominent. Our finding that thermosteric contributions have increased relative to earlier periods is consistent with the acceleration of NIO thermosteric sea level rise from 0.68 mm/yr (1958–2015) to 2.3 mm/yr (1993–2015) documented by Swapna et al.^32^, suggesting that the monsoon-driven heat retention mechanism they identified has continued or intensified during our study period (2003–2024). Spatial trend maps for the steric sea level components derived from the ARMOR3D and IAP datasets are presented in Figure S1, while the corresponding ocean mass trends from the JPL, CSR, and GSFC mascon products are illustrated in Figure S2. Additionally, the regional, area-averaged trends for each of these sea level components are summarized in Table S2.

### Temporal variability of sea level components in the NIO

**Fig. 3:**
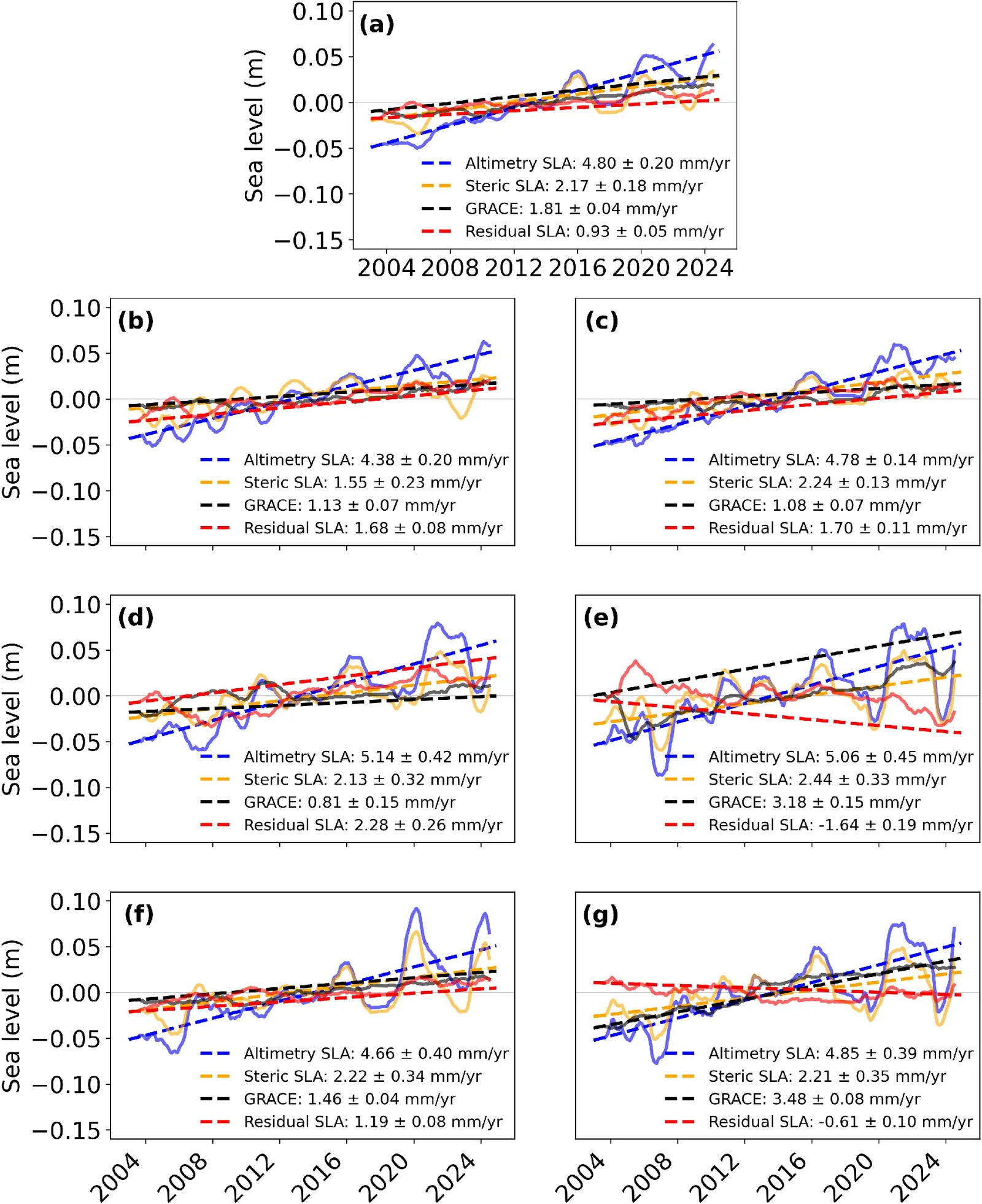
De-seasonalized monthly time series and associated trends (mm/yr) of altimetry SLA, steric sea level (ensemble mean of 2000 m depth-integrated ARMOR3D and IAP temperature and salinity datasets), GRACE ocean mass sea level component (ensemble mean of CSR, JPL and GSFC datasets), and residual sea level from the period 2003 to 2024, estimated using Mann-Kendall and Sen’s slope estimator test over **(a)** NIO, **(b)** WAS, **(c)** EAS, **(d)** WBoB, **(e)** EBoB, **(f)** WEIO, and **(g)** EEIO.

The de-seasonalized monthly time series of major sea level components demonstrate that steric sea level variability generally tracks the altimetry-derived sea level pattern across all NIO sub-basins (Fig. 3). However, in the Arabian Sea, Western Bay of Bengal (WBoB) and Western Equatorial Indian Ocean (WEIO) sub-basins, the contribution of residual sea level is significant. Across the entire NIO (Fig. 3a), the residual sea level contributes approximately 0.93 mm/yr to the total sea level rise, while the steric component alone accounts for about 2.17 mm/yr. In the WBoB (Fig. 3d), the residual component is more prominent (~ 2.28 mm/yr) whereas the EBoB (Fig. 3e) is dominated by the ocean mass component (~ 3.18 mm/yr). However, the EEIO also exhibits higher trends in the ocean mass component (3.48 mm/yr) possibly arising from uncertainties associated with the crustal response to the Sumatra-Andaman earthquake. This partitioning is broadly compatible with earlier basin-scale estimates of steric versus total trends from Salim et al.^35^, but our longer record, comparatively deeper depth and focus on sub-basin scales reveal larger contrasts between west and east, especially in the equatorial Indian Ocean.

**Fig. 4:**
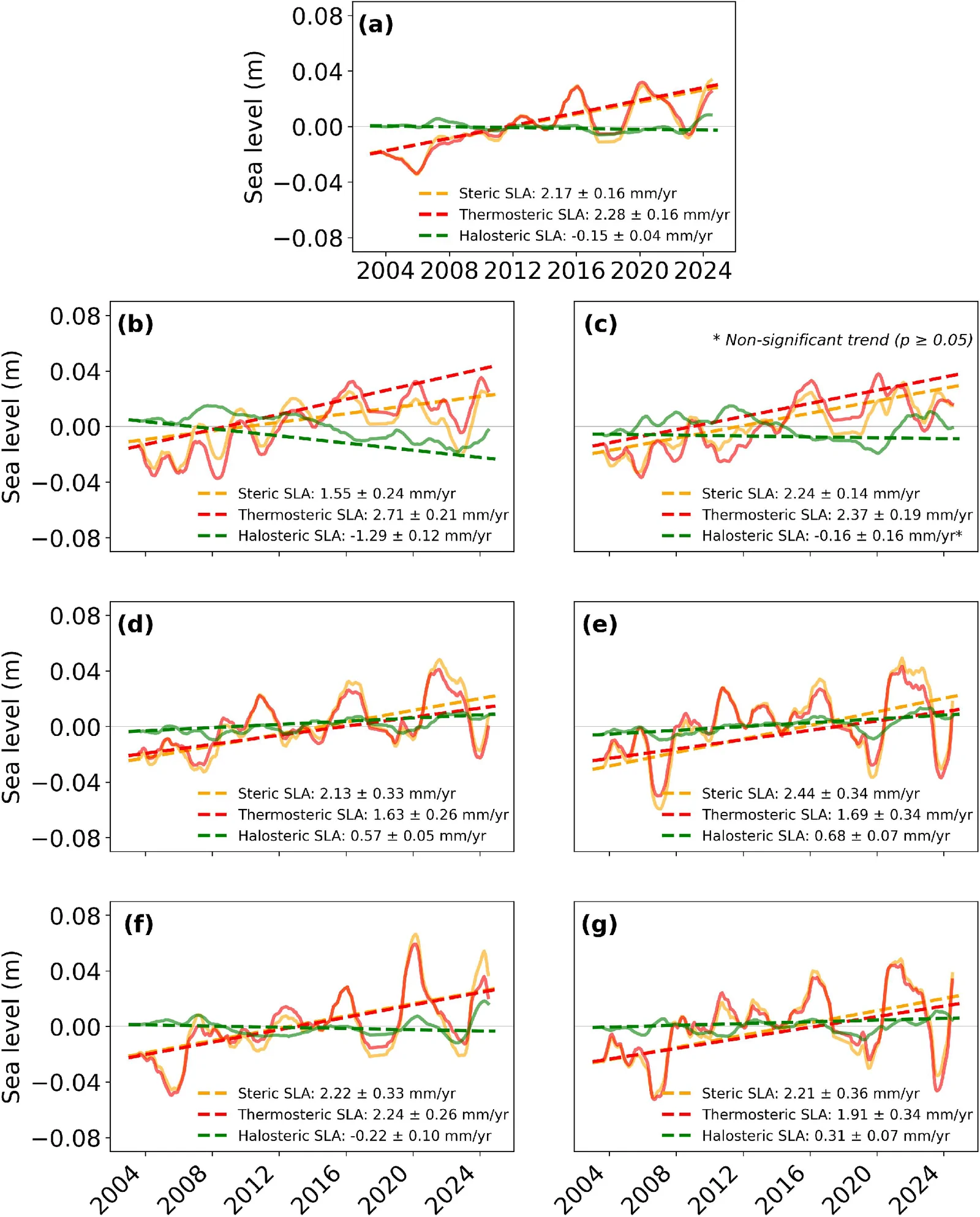
De-seasonalized monthly time series and associated trends (mm/yr) of steric, thermosteric, and halosteric sea level components (ensemble mean of 2000 m depth-integrated ARMOR3D and IAP temperature and salinity datasets) from the period 2003 to 2024, estimated using Mann-Kendall and Sen’s slope estimator test over **(a)** NIO, **(b)** WAS, **(c)** EAS, **(d)** WBoB, **(e)** EBoB, **(f)** WEIO, and **(g)** EEIO.

Since steric sea level change includes both thermosteric and halosteric variations, Fig. 4 depicts these individual contributions. In the NIO, all the sub-basins except in the WAS (Fig. 4b), there is an increasing trend observed in steric sea level, which is ranging from 2.13 mm/yr in WBoB (Fig. 4d) to 2.44 mm/yr in the EBoB (Fig. 4e). Thermosteric effects emerge as the leading driver for accelerating the sea level trends in the NIO (2.28 mm/yr) and most of its sub-basins. However, a strong negative halosteric trend (−1.29 mm/yr) in the WAS is partially offsetting the strong positive thermosteric trend (2.71 mm/yr) in the region. High salinity outflows from the Persian Gulf and Red Sea have caused elevated salinity in the Arabian Sea. This elevated salinity enhances saline contraction and strengthens the negative halosteric sea level changes^71,72^. In contrast, the Bay of Bengal (WBoB and EBoB) exhibits significant positive halosteric trends, 0.57 mm/yr in WBoB and 0.68 mm/yr in EBoB, driven by increased freshwater input from river discharges. This freshening effect plays a major role in accelerating the total steric sea level variability in the BoB region. A similar contrast between Arabian Sea salinification and BoB freshening has also been noted in steric analyses of the upper Indian Ocean^35^, reinforcing the robustness of this pattern across different datasets and methodologies.

#### Inter-annual variability of sea level and the role of climate modes

Steric variations play a leading role in shaping the temporal evolution of sea level across the North Indian Ocean, with thermosteric effects contributing most significantly to the observed variability (Fig. 4a). While other components of sea level, such as mass-driven and residual contributions, exhibit different patterns than the total sea level from satellite altimetry, steric and thermosteric signals remain closely correlated with the altimetric observations, particularly at interannual timescales. This strong coherence between SSH and steric signals in the NIO is consistent with Salim et al.^35^, who found that the first two complex EOF modes of steric height explained more than 70% of SSH variability over the tropical Indian Ocean.

**Fig. 5:**
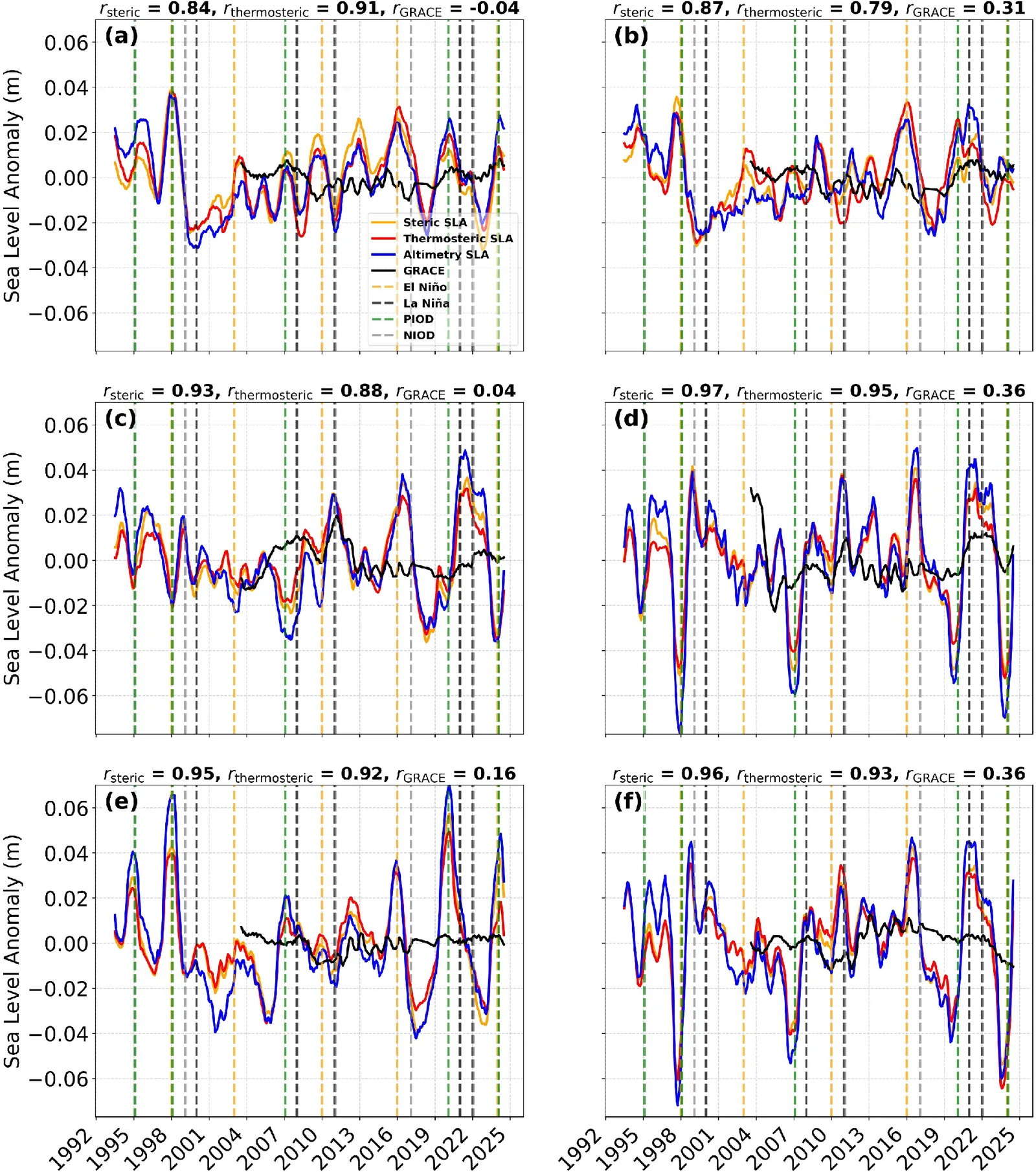
Detrended monthly sea level anomalies of steric, thermosteric, GRACE-derived ocean mass, and altimetry-derived sea level in the following North Indian Ocean sub-basins: **(a)** WAS, **(b)** EAS, **(c)** WBoB, **(d)** EBoB, **(e)** WEIO, and **(f)** EEIO. The altimetry, steric and thermosteric sea level anomalies are shown from 1993 to 2024 whereas the ocean mass component is shown from 2003–2024. Vertical lines indicate the years of occurrence of various climate modes, as detailed in Table 2.

To examine this relationship, further, Fig. 5 compares detrended monthly anomalies of total sea level from altimetry with both steric and thermosteric sea level across the NIO sub-basins. A strong positive correlation, significant at the 99% confidence level, is observed between altimetric sea level and both steric components in all sub-basins. In most sub-basins, steric sea level shows slightly higher correlations with altimetry than the thermosteric component alone. However, in the WAS, thermosteric sea level anomalies exhibit a stronger correlation (*r* = 0.91) with altimetric sea level than the total steric signal (*r* = 0.84), emphasizing the dominant role of temperature in driving sea level variability in this basin. This thermosteric dominance in the Arabian Sea at interannual timescales is consistent with the findings of Srinivasu et al.^34^, who showed that the upper 700 m thermosteric sea level explains approximately 94% of observed NIO sea level rise during their Period II (2004–2013), with particularly strong thermosteric control in the Arabian Sea. However, the influence of the ocean mass component on the interannual variability of total sea level is observed to be less significant in the sub-basins.

To further assess the impact of large-scale climate modes on sea level variability, vertical dashed lines representing years of major climate events were added to Fig. 5. These include the strongest 1997-98 El Niño, the 2010-11 La Niña, and 2019-20 positive Indian Ocean Dipole (pIOD) event (see Table 2). These periods exhibit clear sea level anomalies, especially in the BoB and the Equatorial Indian Ocean, indicating that strong ENSO and IOD events significantly influence regional sea level patterns. These variations could be primarily linked to large-scale planetary waves like Kelvin waves moving eastward and Rossby waves moving westward, which dominate the first mode of sea level anomaly patterns^73^. Equatorial wind patterns play a key role by generating these Kelvin waves that then influence sea levels across the ocean basin^74^. The Indian Ocean Dipole (IOD) primarily affects regional climate, while ENSO influences global weather patterns, including impacts on the Indian Ocean through atmospheric circulation changes^75^. Both ENSO and IOD significantly drive sea level fluctuations in the Eastern Indian Ocean^76^.

**Table 2:** Classification of IOD and ENSO years based on Relative Oceanic Niño Index (RONI) and Dipole Mode Index (DMI) obtained from NOAA Physical Sciences Laboratory.

Events	Years
pIOD	1994, 1997, 2006, 2019, 2024 (all strong events)
El Niño	1994-95 1997-98 (strong) 2002-03 2006-07 2009-10 (strong) 2015-16 (strong) 2023-24 (strong)
nIOD	1998, 2010, 2016, 2021, 2022 (all strong events)
La Niña	1998-99, 2007-08, 2010-11, 2020–2022 (all strong events)

**Fig. 6:**
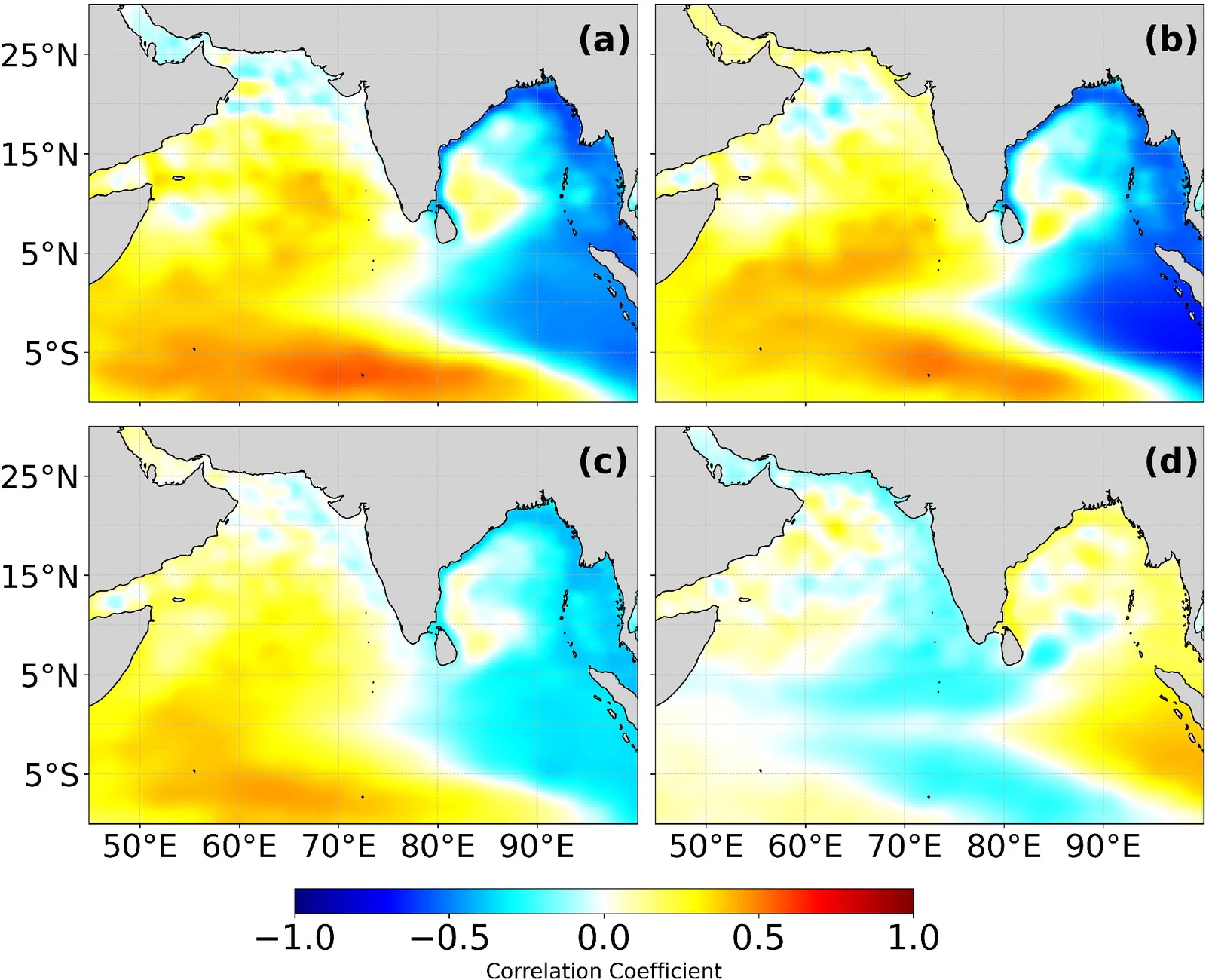
Spatial correlation maps of monthly mean SLA with: **(a)** Relative Oceanic Niño Index (RONI), **(b)** Dipole Mode Index (DMI), **(c)** Western Tropical Indian Ocean (WTIO) SST index, **(d)** Southern Tropical Indian Ocean (SETIO) SST index for the period 1993–2024.

To better understand the spatial footprint of these climate drivers, Fig. 6 presents spatial correlation maps between sea level anomalies and key climate indices, including the Oceanic Niño Index (ONI), Dipole Mode Index (DMI), and SST anomalies in the Western Tropical Indian Ocean (WTIO) and Southeastern Tropical Indian Ocean (SETIO). DMI, which is calculated as the difference between WTIO and SETIO SSTs^77^, offers insight into the spatial structure of IOD-related sea level responses. The spatial correlation patterns between sea level and the ONI and DMI exhibit distinct, though partially concurrent, structures across the NIO. A consistent response to both climatic indices is observed, with positive correlations prevalent in the western NIO and negative correlations dominating the eastern basin, the latter of which extends zonally along the equatorial belt to nearly 75°E. In the BoB, the two indices share a dipole-like correlation pattern, wherein a modest positive signal in the southwest contrasts with widespread negative correlations elsewhere in the basin. These are consistent with the findings of Nidheesh et al.^37^, who demonstrated that at interannual timescales, IOD events drive sea level variations in the Eastern Equatorial Indian Ocean and BoB through equatorial and coastal waveguide dynamics, with Kelvin waves propagating from the central Indian Ocean to the eastern boundary and subsequently into the BoB as coastal Kelvin waves.

In the equatorial Indian Ocean, the correlation fields reveal a dipole configuration similar to that observed in the BoB, with both indices registering robust positive values (*r* > 0.5) in the WEIO and substantial negative values in the EEIO. A salient inter-index difference, however, is the greater magnitude of the negative DMI-sea level correlation (*r* < −0.5) relative to the ONI-sea level correlation (*r* ≥ −0.3) over the eastern sector of the basin. Correlations with the WTIO and SETIO SST indices provide further clarity. The WTIO SST index shows a spatial correlation structure that closely mirrors that of the DMI and ONI, underscoring its dominant role in regulating sea level variability across the region. This structural resemblance highlights WTIO’s mediating influence on the DMI-sea level relationship, particularly over the Equatorial Indian Ocean and BoB. Furthermore, as demonstrated by Tong et al.^78^, rising local temperatures and persistent easterly wind anomalies in the western basin serve as robust early indicators, offering considerable potential for enhancing the lead-time predictability of pIOD events. Such dipole-like patterns, and their non-stationarity over multidecadal time scales, are in line with the evolving spatial trend structures inferred from reconstructed sea level fields in the Indian Ocean by Palanisamy et al.^24^. Ghomsi et al.^7^ similarly found that remote climate drivers, including ENSO and the IOD, modulate sea level variability across the Somali Coastal Current and Red Sea LMEs, with regression coefficients of up to 24 mm for the DMI, underscoring the basin-wide reach of these teleconnections.

**Fig. 7:**
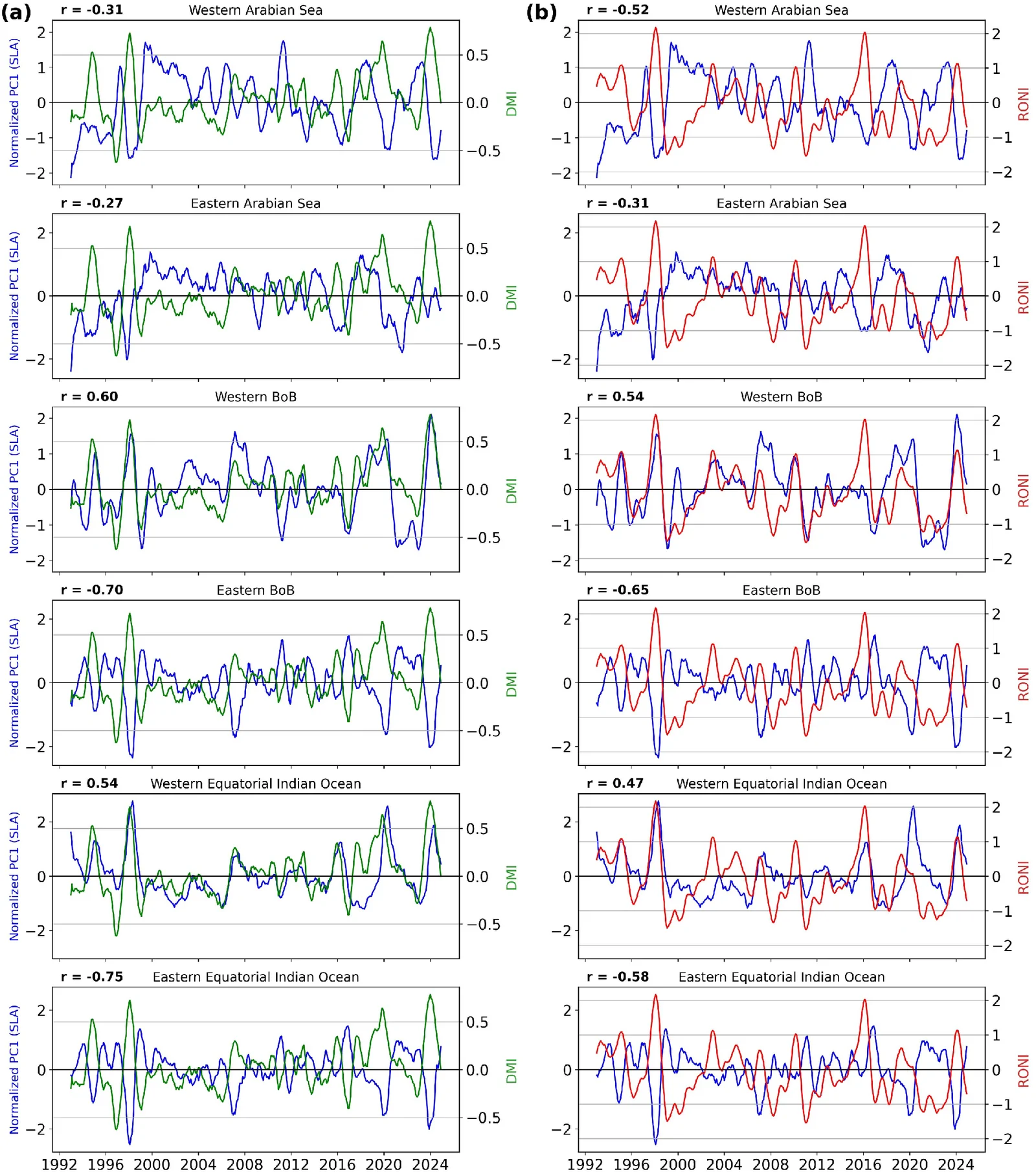
First Principal Component (PC1) of altimetry SLA and its comparison with **(a)** DMI and **(b)** RONI across various sub-basins of the NIO during 1993–2024.

Figure 7 illustrates the first principal component (PC1) of the leading empirical orthogonal function (EOF) mode of altimetry-derived sea level anomalies (SLA), alongside the Dipole Mode Index (DMI) and Oceanic Niño Index (ONI), to examine regional interannual sea level variability across the NIO. As PC1 captures the dominant mode of SLA variability, the extent to which large-scale climate modes influence sea level dynamics within specific sub-basins can be assessed by comparing it with major climate indices. A notable feature in the PC1 patterns is a dipole structure between the western and eastern sub-basins of both the Bay of Bengal (WBoB and EBoB) and the Equatorial Indian Ocean (WEIO and EEIO), with opposing phase variability in sea level between these sectors. This phase variability along with stronger correlation is less evident in the EAS, suggesting that the interannual sea level dynamics in that region is less sensitive to ENSO or IOD forcing. Comparative analysis reveals that DMI exhibits a stronger correlation with PC1 across most sub-basins than ONI, particularly in the BoB and Equatorial Indian Ocean.

The Western sub-basins of both the BoB and Equatorial Indian Ocean show PC1 variability that closely tracks phases of the DMI and, to a lesser extent, the ONI, while eastern sub-basins show opposite-phase behavior. This anti-phase response suggests a coherent basin-wide adjustment of sea level anomalies to the shifting patterns of climate forcings^79^. Palanisamy et al.^24^ similarly highlighted, that Indian Ocean Sea level exhibits east-west dipole structures strongly correlated with IOD events, which our analysis confirms for the more recent 1993–2024 period at sub-basin scales.

During strong positive IOD (pIOD) events or combined pIOD-El Niño years, western sub-basins of BoB and EIO generally exhibit increased sea level, whereas eastern sub-basins tend to show negative anomalies. Conversely, strong negative IOD (nIOD) events or combined nIOD and La Niña years produce the opposite effect, with elevated sea level in the eastern sub-basins and reductions in the western sub-basins. This spatial signature matches previously reported patterns of sea level change associated with ENSO and IOD phases^18,80^. During pIOD events, strong easterly winds replace the usual westerlies over the equatorial Indian Ocean, causing sea levels to drop in the eastern basin. These easterlies generate upwelling Kelvin waves that travel eastward, reflect off the coast, and propagate back as Rossby waves, further lowering sea levels, or propagate as coastal trapped Kelvin waves that move poleward^81^. The easterly wind anomalies also generate an equatorial-downwelling Rossby wave, which reflects off the western boundary as a downwelling Kelvin wave. This wave propagation leads to elevated sea levels in the WEIO^82–84^. Nidheesh et al.^37^ demonstrated that this wave-mediated teleconnection results in high correlations (*r* > 0.8) between EEIO and BoB Sea level at both interannual and decadal timescales, while the southwestern Indian Ocean region is more weakly correlated with eastern basin variability at decadal timescales due to the independent influence of southern Indian Ocean wind stress curl. At such times, the usual downwelling Kelvin waves, which typically elevate sea levels in the eastern BoB, are significantly weakened or absent. Instead, only upwelling-favourable Kelvin waves dominate, generating Rossby waves along the eastern BoB rim that further reduce sea levels. In contrast, normal years feature stronger downwelling Kelvin waves, leading to higher sea levels in this region^81^. Meanwhile, the southwestern BoB shows higher sea levels during pIOD/El Niño events due to wind-driven Ekman pumping, where easterly anomalies create an anticyclonic (clockwise) circulation that elevates the sea level^76^. Thus, IOD/ENSO phases produce contrasting sea level responses in the BoB.

However, not all strong El Niño years lead to significant SLA changes in the NIO. For instance, the 2002–2003 El Niño, despite being a moderate-intensity event, did not result in notable SLA variations across the region. In contrast, the strongest positive IOD event in 2019 during a weaker El Niño period produced pronounced sea level rise in the eastern sub-basins and significant decreases in the western counterparts, as shown in Fig. 7. These patterns highlight the asymmetric and regionally specific responses of NIO sea level to different modes of climate variability. The results are in agreement with previous studies that emphasize the modulation of interannual sea level variability in the NIO by IOD and ENSO-related processes^33,36^.

## Summary and conclusions

This study quantifies the relative contributions of different sea level components to total sea level trends in the North Indian Ocean during 2003–2024 when the GRACE gravimetry measurements of the mass-driven ocean mass component were available. Six sub-basins were analyzed, covering the Arabian Sea, BoB, and Equatorial Indian Ocean. A key methodological finding is the strong agreement between the parametric Ordinary Least Squares (OLS) trends and the non-parametric Mann-Kendall/Sen’s slope estimates. This consistency shows that the observed sea level variations are stable, long-term trends, and also confirms that these results are not distorted by short-term climate anomalies or single-year data extremes at the ends of the time series. The North Indian Ocean (NIO) represents a region with complex dynamics for the sea level budget. Unlike the global mean sea level, which is primarily mass-driven, trends in the NIO are predominantly governed by thermosteric sea level rise, consistent with the accelerated regional warming observed over recent decades. This thermosteric dominance confirms and extends the findings of Swapna et al.^32^, who demonstrated that weakening Indian summer monsoon circulation drives increased heat retention in the NIO through reduced southward heat transport via the Cross-Equatorial Cell, and of Srinivasu et al.^34^, who documented the onset of accelerated NIO sea level rise after 2003. Spatial trend analysis reveals a significant positive sea level trend across the NIO (4.83 ± 0.22 mm/yr), with the thermosteric component dominating the regional average. This rate exceeds the 1993–2023 global mean of ~ 3.4 mm/yr and is comparable to the accelerated rates (~ 4.3 mm/yr since 2010) reported for African coastal waters by Ghomsi et al.^7^, indicating that the NIO is experiencing similarly intensified sea level rise. At the basin scale, steric contribution (2.23 ± 0.18 mm/yr) accounts for roughly 46% of the total observed sea level rise, driven almost entirely by thermal expansion (2.21 ± 0.16 mm/yr). The halosteric contribution is negligible (−0.10 ± 0.05 mm/yr), however, the mass-driven contribution (GRACE: 1.69 ± 0.09 mm/yr) accounts for roughly 35% of the observed rise, confirming that a substantial amount of mass is being redistributed into the basin. Sub-basin differences emerge however. The Eastern Equatorial Indian Ocean (EEIO) exhibits a mass-dominated trend (3.42 ± 0.11 mm/yr), more than 1.5 times the thermosteric contribution. Halosteric influences differ sharply across basins, where, the Arabian Sea exhibits strong negative trends (−0.71 mm/yr), while the Bay of Bengal (BoB) sub-basins show positive halosteric contributions (~ 0.60 mm/yr), linked to freshwater input. A similar halosteric suppression of sea level rise has been documented in the Mediterranean by Ghomsi et al.^7^, where increased salinity offsets thermosteric expansion, highlighting the importance of salinity-driven density changes in semi-enclosed and marginal seas.

Regional differences in ocean mass trends appear to be shaped by distinct processes. For example, the 2004 Sumatra-Andaman earthquake likely contributed to the sharp trend variations in the EEIO, while in the BoB, the signals of earthquake along with other factors including river discharge and sediment transport plays more dominant role. Basin-level decomposition reveals contrasting drivers where thermosteric changes dominate sea level trends in the Arabian Sea, while the EIO shows a west-east gradient, transitioning from thermosteric-dominated in the west to ocean mass component dominated in the east. In the BoB, the steric contribution in both the sub-basins are 2.3 mm/yr. However, uncertainties in the steric sea level measurements could be introduced by the absence of trend data near the northern and eastern Bay of Bengal coasts, in addition to limitations associated with the ocean mass trends from the GRACE dataset in the Andaman-Sumatra region of Eastern Indian Ocean. Taken together with previous Indian Ocean studies^24,27,35^ and the pan-African synthesis of Ghomsi et al.^7^, our results indicate that recent decades have seen both an acceleration of NIO sea level rise and an increasing fractional contribution from thermosteric processes, while mass and residual terms remain highly heterogeneous and locally important.

Interannual variability in NIO sea level is primarily governed by thermosteric fluctuations, which show strong spatial and temporal agreement with altimetry-derived anomalies. This consistency highlights the influence of regional warming and temperature variability on total sea level change. Climate modes, particularly ENSO and the Indian Ocean Dipole (IOD), display strong correlations with sea level, but their effects vary spatially. Western sub-basins of the BoB and Equatorial Indian Ocean exhibit in-phase relationships with these climate indices, whereas eastern sub-basins show anti-phase responses, resulting from changes in wind anomalies and dynamics related to the long-period waves associated with the climatic events. The Eastern Arabian Sea appears less affected by these large-scale climate modes. Srinivasu et al.^34^ similarly found that decadal sea level variability in the NIO is primarily controlled by surface wind stress changes over the Indian Ocean, with limited influence from the Pacific through the Indonesian Throughflow, consistent with our finding that Arabian Sea sea level is less responsive to ENSO/IOD forcing. Analysis of the Western Tropical Indian Ocean (WTIO) and Southeastern Tropical Indian Ocean (SETIO) SST indices further shows that WTIO captures the Dipole Mode Index (DMI) pattern and is a strong predictor of regional sea level variability. Since the spatial correlation structure of the WTIO SST index closely mirrors the full DMI, it is evident that sea surface temperature fluctuations in the western tropical boundary act as a major mediator for sea level changes across the NIO. This reinforces earlier conclusions that IOD-related variability is a primary driver of Indian Ocean regional sea level changes on interannual time scales^24,35^, and aligns with the finding of Ghomsi et al.^7^ that Atlantic and Indian Ocean climate modes (including ENSO and IOD) modulate regional sea level anomalies through SST-driven thermal expansion and circulation changes.

In summary, the total sea level trend in the NIO is 4.83 mm/yr, with thermosteric sea level driven by ocean warming emerges as the dominant contributor, also influencing interannual variability. However, regional contributions vary significantly, with mass changes, halosteric effects, and residual processes also playing important roles in specific sub-basins. In contrast to African LMEs, where mass contributions now dominate^7^, the NIO remains a thermosteric-dominated system, although the EEIO and the EBoB exhibit substantial mass-driven sea level trends that warrant further investigation. Future research should prioritize deeper investigation into the mass-driven uncertainties in the eastern Indian Ocean as well as the residual sea level component, which may reflect unmodelled processes such as sediment loading, vertical land motion, or regional hydrology, and remains a key source of uncertainty in regional sea level budgets. Combining our sub-basin-scale budget with independent constraints on vertical land motion (as in Palanisamy et al.^24^) and deeper-water steric estimates (beyond 700–900 m; cf. Ghosh et al.^27^), and placing NIO trends in the context of broader Indian Ocean and African coastal changes^7^, would be logical next steps toward closing the NIO sea level budget and distinguishing climatic from anthropogenic land-based contributions.

## Supplementary Information

Below is the link to the electronic supplementary material.

Supplementary Material 1

## Data Availability

All data supporting the results of this study is available in the paper.
