# Supplementary material for: Sub-basin sea level budget analysis in the North Indian Ocean (2003–2024)
Source: Sci Rep. 2026 Jul 5;16:28366. doi: 10.1038/s41598-026-60856-5 (PMC13562562; doi:10.1038/s41598-026-60856-5)
Supplement: Supplementary file 1 [file 41598_2026_60856_MOESM1_ESM.docx]

**Sub-basin sea level budget analysis in the North Indian Ocean (2003-2024)**

Ullas M. Pillai^1,2^, Franck Eitel Kemgang Ghomsi^3,4^, Ajith Joseph Kochuparampil^1^*, Roshin P. Raj^5^ and Ola M. Johannessen^6^

^1^Nansen Environmental Research Centre (India), Kochi - 682506, India ^2^Faculty of Ocean Science and Technology, Kerala University of Fisheries and Ocean Studies, Kochi - 682506, India. [ullasmpillai007@gmail.com](mailto:ullasmpillai007@gmail.com)

^3^Centre for Earth Observation Science, University of Manitoba, Winnipeg, MB, Canada

^4^Nansen-Tutu Centre for Marine Environmental Research, Department of Oceanography, University of Cape Town, South Africa, [franckeitel@gmail.com](mailto:franckeitel@gmail.com)

^5^Nansen Environmental and Remote Sensing Center and Bjerknes Center for Climate Research, Bergen, Norway. [Roshin.Raj@nersc.no](mailto:Roshin.Raj@nersc.no)

^6^Nansen Scientific Society, Bergen, Norway. [ola.johannessen@nansenscientificsociety.no](mailto:ola.johannessen@nansenscientificsociety.no)

***Corresponding author:** Ajith Joseph Kochuparampil, [ajith_jk@nerci.in](mailto:ajith_jk@nerci.in)

**Supplementary materials**

**Table S1**. Area-averaged trends of sea level and its components across the North Indian Ocean (NIO) and its sub-basins from 2003 to 2024 (units in mm/yr) estimated using Ordinary Least Square (OLS) method. The Total SLA consists of the entire altimetry trend from 2003 to 2024, whereas for the remaining components, the sea level trend corresponding to the time period of GRACE dataset (including the missing periods) from 2003 to 2024 is considered. (*Non-significant trends)

| **Region** | **Sub-basin** | **Total Altimetry SLA (mm/yr)** | **Altimetry SLA (mm/yr)** | **Steric (mm/yr)** | **Thermosteric (mm/yr)** | **Halosteric (mm/yr)** | **GRACE (mm/yr)** | **Residual (mm/yr)** |
| --- | --- | --- | --- | --- | --- | --- | --- | --- |
| Arabian Sea | WAS | 4.12 ± 0.17 | 4.23 ± 0.17 | 1.39 ± 0.17 | 2.52 ± 0.16 | -1.13 ± 0.07 | 1.14 ± 0.08 | 1.68 ± 0.10 |
|  | EAS | 4.66 ± 0.19 | 4.83 ± 0.18 | 2.11 ± 0.14 | 2.31 ± 0.17 | -0.20 ± 0.09 | 1.12 ± 0.08 | 1.68 ± 0.11 |
| Bay of Bengal | WBoB | 4.91 ± 0.27 | 5.04 ± 0.27 | 2.07 ± 0.22 | 1.52 ± 0.20 | 0.55 ± 0.05 | 0.84 ± 0.10 | 2.10 ± 0.15 |
|  | EBOB | 4.60 ± 0.41 | 4.60 ± 0.43 | 2.01 ± 0.33 | 1.39 ± 0.30 | 0.62 ± 0.06 | 2.82 ± 0.14 | -1.45 ± 0.16 |
| Equatorial Indian Ocean | WEIO | 4.73 ± 0.31 | 4.96 ± 0.31 | 2.32 ± 0.26 | 2.28 ± 0.23 | 0.04 ± 0.07* | 1.42 ± 0.07 | 1.21 ± 0.09 |
|  | EEIO | 4.59 ± 0.37 | 4.59 ± 0.39 | 1.99 ± 0.31 | 1.71 ± 0.33 | 0.28 ± 0.05 | 3.44 ± 0.08 | -0.52 ± 0.13 |
| NIO | NIO | 4.62 ± 0.15 | 4.76 ± 0.14 | 2.05 ± 0.13 | 2.09 ± 0.12 | -0.04 ± 0.04* | 1.70 ± 0.06 | 0.99 ± 0.06 |

**Figure S1**. Spatial Mann-Kendall trends of SLA (mm/yr) from 2003 to 2024 of Steric (**a, b**), Thermosteric (**c, d**), and Halosteric (**e, f**) sea level from ARMOR3D and IAP datasets. Regions where trends are not statistically significant at the 95% confidence level are stippled.

**Figure S2**. Spatial Mann-Kendall trends of GRACE ocean mass component (mm/yr) for the period 2003 to 2024 from (**a**) CSR, (**b**) JPL and (**c**) GSFC datasets. Regions where trends are not statistically significant at the 95% confidence level are stippled.

**Table S2**. Area-averaged trends (Mann-Kendall) of GRACE ocean mass component and steric components of sea level from various datasets across the North Indian Ocean (NIO) and its sub-basins from 2003 to 2024 (units in mm/yr). (*Non-significant trends)

| **Region** | | **Arabian Sea** | | **Bay of Bengal** | | **Equatorial Indian Ocean** | | **NIO** |
| --- | --- | --- | --- | --- | --- | --- | --- | --- |
| **Sub-basin** | | **WAS** | **EAS** | **WBoB** | **EBoB** | **WEIO** | **EEIO** |  |
| **Steric**  **(mm/yr)** | **ARMOR3D** | 1.19 ± 0.27 | 1.88 ± 0.22 | 1.82 ± 0.41 | 2.13 ± 0.46 | 1.60 ± 0.35 | 1.91 ± 0.47 | 1.90 ± 0.21 |
|  | **IAP** | 1.48 ± 0.25 | 1.99 ± 0.21 | 2.32 ± 0.37 | 2.29 ± 0.41 | 2.23 ± 0.34 | 2.49 ± 0.40 | 2.2 ± 0.20 |
| **Thermosteric**  **(mm/yr)** | **ARMOR3D** | 2.24 ± 0.29 | 2.08 ± 0.25 | 1.39 ± 0.36 | 1.50 ± 0.40 | 1.71 ± 0.32 | 1.79 ± 0.44 | 1.93 ± 0.16 |
|  | **IAP** | 2.70 ± 0.25 | 2.20 ± 0.24 | 1.66 ± 0.32 | 1.69 ± 0.36 | 2.16 ± 0.29 | 2.24 ± 0.38 | 2.24 ± 0.15 |
| **Halosteric**  **(mm/yr)** | **ARMOR3D** | -1.12 ± 0.12 | -0.34 ± 0.17 | 0.46 ± 0.08 | 0.61 ± 0.10 | -0.31 ± 0.10 | 0.14 ± 0.08 | -0.20 ± 0.05 |
|  | **IAP** | -1.23 ± 0.13 | -0.31 ± 0.12 | 0.67 ± 0.07 | 0.61 ± 0.08 | -0.10 ± 0.11* | 0.30 ± 0.07 | -0.03 ± 0.05* |
| **GRACE**  **(mm/yr)** | **CSR** | 0.50 ± 0.15 | 0.80 ± 0.19 | 0.60 ± 0.19 | 3.68 ± 0.28 | 1.36 ± 0.13 | 4.34 ± 0.13 | 1.70 ± 0.13 |
|  | **JPL** | 1.19 ± 0.10 | 0.61 ± 0.11 | 0.62 ± 0.14 | 3.47 ± 0.20 | 1.51 ± 0.10 | 3.58 ± 0.10 | 1.80 ± 0.07 |
|  | **GSFC** | 1.61 ± 0.14 | 1.75 ± 0.16 | 1.14 ± 0.18 | 1.59 ± 0.27 | 1.32 ± 0.13 | 2.29 ± 0.15 | 1.51 ± 0.12 |
